# Supplementary material for: The interactome of intact mitochondria by cross-linking mass spectrometry provides evidence for coexisting respiratory supercomplexes
Source: Mol Cell Proteomics. 2017 Dec 8;17(2):216–32. doi: 10.1074/mcp.RA117.000470 (PMC5795388; doi:10.1074/mcp.RA117.000470)
Supplement: Supplemental Data [file supp_RA117.000470_133922_0_supp_23978_fzffwf.zip › spectra_annotation/mito_spectra_annotation/337-1-3-1-7-1.pdf]

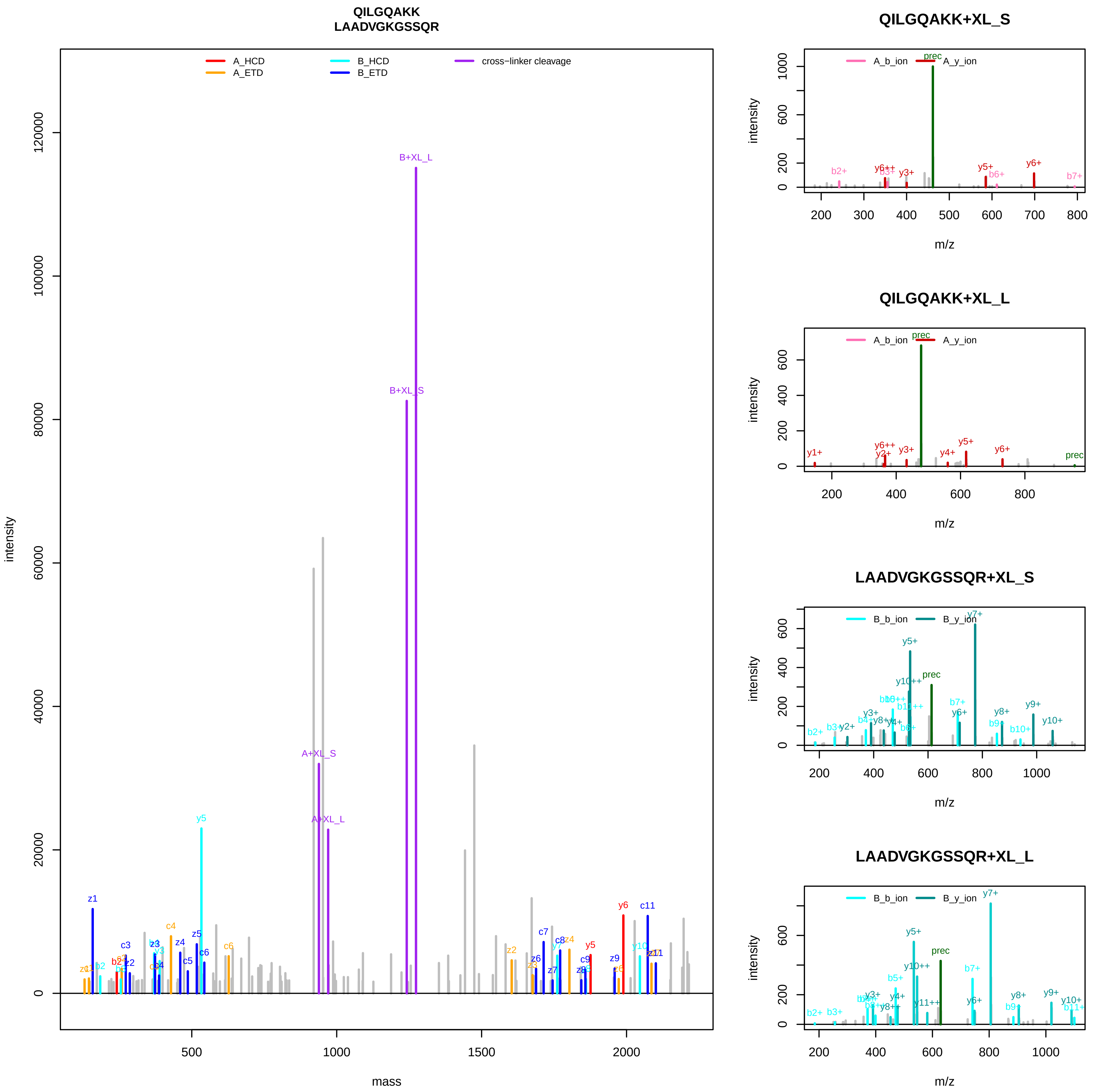

QILGQAKK
LAADVGKGSSQR
A_HCD
A_ETD
B_HCD
B_ETD
cross−linker cleavage
B+XL_L
B+XL_S
A+XL_S
A+XL_L
y5
z1
c3
z3
c4
z4
z5
c5
c6
c7
c8
z4
y5
y6
c11
z9
z2
z6
z7
y10
intensity
mass
0
20000
40000
60000
80000
100000
120000
500
1000
1500
2000
QILGQAKK+XL_S
A_b_ion
A_y_ion
prec
b2+
y6++
y3+
y5+
b6+
y6+
b7+
m/z
QILGQAKK+XL_L
y1+
y6++
y2+
y3+
y4+
y5+
y6+
LAADVGKGSSQR+XL_S
B_b_ion
B_y_ion
y7+
y5+
y10++
b2+
b3+
y2+
y3+
y8+
b7+
y6+
y8+
y9+
y10+
b10+
LAADVGKGSSQR+XL_L
y7+
y5+
prec
y10++
b7+
y6+
y8+
y9+
y10+
y11++
